# Investigation of Antiphosphatidyl-Serine Antibody and Antiphosphatidyl-Inositol Antibody in Ischemic Stroke Patients

**DOI:** 10.1155/2010/439230

**Published:** 2010-12-20

**Authors:** Hirohisa Okuma, Yasuhisa Kitagawa, Shigeharu Takagi

**Affiliations:** ^1^Department of Neurology, Tokai University Tokyo Hospital, Tokyo 151-0053, Japan; ^2^Department of Neurology, Tokai University Hachioji Hospital, Tokyo 192-0032, Japan; ^3^Department of Neurology, Tokai University School of Medicine, Kanagawa 259-1193, Japan

## Abstract

Antiphospholipid syndrome is characterized by arterial or venous thrombosis and the presence of antiphospholipid antibodies (aPL). We measured *β*2-GPI aCL, IgGaCL, LA, antiphosphatidyl-serine antibody (PS), and antiphosphatidyl-inositol antibody (PI) in each patient at one month after the onset of stroke. In addition, carotid artery echography was performed in patients positive for PI or PS. Among the 250 patients, 13.6% (34/250) were positive for either PI or PS, and 6.8% (17/250) were positive for both. Carotid artery echography performed on these 34 patients showed that the frequencies of increased intimal-medial thickness (IMT) of 1.1 mm or more, plaque, and carotid artery stenosis of 50% or more were all significantly higher in patients positive for antinuclear antibody than those negative for the antibody (*P* < .05). PI and PS are associated with antinuclear antibody and precipitation of atherosclerosis. Ischemic stroke patients with SLE frequently showed a variety of antiphospholipid-protein antibodies.

## 1. Introduction

In cerebrovascular disorders with an underlying immune abnormality, antiphospholipid antibodies, of which there are numerous types, are the leading cause of thrombosis in the absence of acquired risk factors [1]. Cardiolipin was the first identified antigen for antiphospholipid antibodies, and other anionic phospholipids were subsequently recognised as antigens. It is now considered that proteins that bind to phospholipids play an important role in thrombogenesis. Among these proteins, *β*2-glycoprotein is the most important [2–4]. Known antigens to antiphospholipid antibodies include cardiolipin, the anionic phospholipids phosphatidylserine and phosphatidylinositol, and neutral phosphatidylethanolamine. There is also a group of proteins, including prothrombin, annexin V, protein C, protein S, low-molecular-weight kininogen, and factor XI, that bind to phospholipids and are known as antiphospholipid-protein antigens [5]. Recently, the antiprothrombin antibody has been under investigation as a possible new autoantibody. This antibody binds to prothrombin in the presence of cardiolipin and phosphatidylserine [6]. To define and characterise these antibodies, we studied their prevalence rates in patients who had experienced cerebral infarction, and investigated the relationships among the antibodies.

## 2. Materials and Methods

This study involved 250 patients, 155 males and 95 females (average age 72 years), with cerebral infarction who visited our hospitals. Of these patients, one male and four females (average age 39 years) had systemic lupus erythematosus (SLE) as an underlying disease. Levels of antiphospholipid antibodies, including *β*2-glycoprotein I-dependent anticardiolipin antibody (*β*2-GPI aCL), IgG anticardiolipin antibody (IgG aCL), lupus anticoagulant (LA), antiphosphatidyl-serine antibody (PS), and antiphosphatidyl-inositol antibody (PI), were determined in the 250 patients. The level of antinuclear antibody was also measured in patients positive for PI or PS. Measurement of PI and PS is an enzyme-linked immunosorbent assay (ELISA) using a solid-phase method. More specifically, 100 *μ*L of L-*α*-phosphatidyl-L-serine or L-*α*-phosphatidyl-L-inositol and the 50-fold diluted serum to be tested are added to microplates preprocessed with 5 *μ*g/mL of protamine sulfate to allow them to react for 90 minutes at 37°C. A buffer (0.01 MPBS and 0.05% Tween) is used to clean unreacted substances, to which 100 *μ*l of peroxidase-labelled antihuman IgG antibody is then added to allow them to react for 90 minutes at room temperature. A buffer is used to clean unreacted labelled antibodies, to which 0.4 mg/mL of o-phenylenediamine solution with 0.012% hydrogen peroxide added is then dispensed in 100-*μ*L aliquots to leave them standing for 20 minutes at room temperature. After that, 2.5 M sulfuric acid was added to them to stop the reaction for the measurement of their absorbance at 490 nm. Results were expressed as the cut-off index, which is equal to the ratio of the absorbance of the serum to be tested to that of a healthy person, with an absorbance of 1.0 or more defined as positive. In addition, carotid artery echography was performed in patients positive for PI or PS. Assessment of the presence of various antibodies was carried out in patients in the chronic stage, at least 1 month after the onset of cerebral infarction. This study was analyzed statistically using *t*-test.

## 3. Results

The prevalence rates of the antibodies in the 250 patients with cerebral infarction. They were 2.8% (7/250) for *β*2-GPI aCL, 12.0% (30/250) for IgG aCL, 9.2% (23/250) for LA, 9.6% (24/250) for PS, and 8.8% (22/250) for PI. Patients aged 50 years or under accounted for 5.2% of the 250 patients (13/250), and of these, 5 had underlying SLE (1 male and 4 females, average age 39). Of these 5 patients, 80% (4/5) were positive for both *β*2-GPI aCL and LA, and 40% were positive for PI or PS as well as *β*2-GPI aCL or LA. One was negative for *β*2-GPI aCL and LA and positive for PI and PS (Table 1). 

Among the 250 patients, 13.6% (34/250) were positive for either PI or PS, and 6.8% (17/250) were positive for both. Of the 34 patients positive for either PI or PS, 8.8% (3/34) were positive for LA, and 8.8% (3/34) were positive for b2-GPI aCL, with 70.6% (24/34) positive for antinuclear antibody. 

Of the 24 patients positive for antinuclear antibody, 50% (12/24) had lacunar infarction, and 41.2% (10/24) had atherothrombotic cerebral infarction. Of the patients with lacunar infarction, 12.5% (3/24) were positive for PI only, 16.7% (4/24) were positive for PS only, and 20.8% (5/24) were positive for both PI and PS. Of the patients with atherothrombotic cerebral infarction, 4.2% (1/24) were positive for PI only, 4.2% (1/24) were positive for PS only, and 33.3% (8/24) were positive for both PI and PS. The incidence of cardiogenic cerebral embolism in patients positive for antinuclear antibody was 8.3% (2/24): 1 was positive for PS only, and 1 was positive for both PS and PI. One patient was positive for all of PI, PS, *β*2-GPI aCL, LA, and antinuclear antibody, and this patient had atherothrombotic cerebral infarction (Table 2). 

Of the 34 patients positive for PI or PS, 79.2% were positive for antinuclear antibody, suggesting that PI and PS may be related to the antinuclear antibody. Carotid artery echography performed on these 34 patients showed that the frequencies of increased intimal-medial thickness (IMT) of 1.1 mm or more, plaque, and carotid artery stenosis of 50% or more were all significantly higher in patients positive for antinuclear antibody than those negative for the antibody (*P* < .05) (Table 3).

## 4. Discussion

Previous reports have suggested that the presence of antiphospholipid antibody is a risk factor for cerebral infarction in those aged under 45 or 50 years [7]. The Antiphospholipid Antibodies in Stroke Study (APASS) [8] reported that in patients with antiphospholipid antibody, the risk of cerebral infarction was 2.31 times higher than in those negative for the antibody, and Brey et al. [9]. reported a 1.5 times higher risk over an observation period of 20 years. In our study of 250 patients with cerebral infarction, the prevalence rates of the antiphospholipid antibodies *β*2-GPI aCL, LA, PI and PS were higher in patients aged 50 or under with underlying SLE than in those without SLE, suggesting that the presence of antiphospholipid antibody may be a risk factor for juvenile cerebral infarction in SLE patients [10]. Antiphospholipid antibodies include anticardiolipin antibody, LA, and antibodies specific to anionic phospholipids, including PS and PI [11, 12]. As mentioned above, PI and PS were detected in 9.6% and 8.8%, respectively, of the 250 patients with cerebral infarction, and 79.2% of these patients tested positive for antinuclear antibody. Of the 250 patients, there were 13 aged 50 or under (average age 43), 4 of whom were positive for PI and PS antibodies, suggesting that the presence of these antibodies should be determined in order to assess the risk of juvenile cerebral infarction. Tuhrim et al.[11] concluded that the presence of PI is a risk factor for juvenile cerebral infarction, andthe results of our study are consistent with that. And also Blank et al. [13] extracted PS from two APS patients, one with habitual abortion and the other who developed recurrent deep thrombophlebitis three times, and administered it to pregnant mice to observe various parameters. In this experiment, the administration of IgG PS to mice with immature placentas and fetuses within 9 weeks of gestation caused prolonged aPTT, thrombocytopenia, increases in placental death of 40% to 50%, and decreases in the mean weights of placentas and fetuses. Based on the results, they concluded that PS could form APS features independently on an experimental basis and suggested that it was important to check for the presence of PS in actual APS patients even if aCL was negative. It is believed that this report supports our results that PS and PI may be risk factors for juvenile cerebral infarction. Results of carotid artery echography in patients positive for PI or PS suggested that these two antibodies are associated with the promotion of arteriosclerosis. 

No significant difference in the type of cerebral infarction was observed in patients positive for antinuclear antibody, but patients positive for both PI and PS tended to have atherothrombotic cerebral infarction.

Thus, we consider that PS and PI, as well as *β*2-GPI aCL and LA, are important in screening for antiphospholipid antibody syndrome and should be regarded as antibodies associated with cerebral infarction [14]. It remains controversial whether the presence of antiphospholipid antibody is associated with an increased risk of recurrent cerebral infarction. The APASS [15] in 1990 found that the incidence of recurrent cerebral infarction was 9.4% and that of TIA was 6.3% over an average observation period of 1.4 years, while Levine et al. [16] reported in 1992 that cerebral infarction and TIA recurred at a high incidence of 35% over an average observation period of 1.2 years. On the other hand, Tanne et al. [17] suggested that the presence of antiphospholipid antibody is not a risk factor for recurrent cerebral infarction. Further work is needed to resolve the issue, and our follow-up study of recurrent cerebral infarction in patients positive for antiphospholipid antibodies is under way. 

The present results suggest that antiphospholipid antibodies should be regarded as a risk factor for juvenile cerebral infarction and that PS and PI, in addition to *β*2-GPI aCL and LA, should be included in the routine tests conducted in patients with cerebral infarction of unknown cause.

Antiphospholipid antibody is a risk factor for cerebral infarction, especially in SLE patients, and in the younger population. PS and PI, in addition to *β*2-GPI aCL and LA, are important in screening for antiphospholipid antibody syndrome and appear to be associated with cerebral infarction. And antinuclear antibody is detected at higher frequency in patients with cerebral infarction who are positive for PS and PI.

## Figures and Tables

**Table 1:** Antiphospholipid-protein antibodies in stroke patients with SLE.

Case	Age/Gender	*β*2-GPI aCL	IgG-aCL	LA	PI	PS
1	50 M	+	−	+	−	+
2	41 F	+	−	+	−	−
3	29 F	+	+	+	−	−
4	31 F	+	+	+	+	+
5	46 F	−	−	−	+	+

**Table 2:** Clinical and laboratory findings in patients with PI and PS.

Case	Age	Gender	PI	PS	*β*2-GPIaCL	ANA	Type of
	(y)						stroke
1	68	Male	0.5	1.4	(−)	D80×	L
2	63	Male	1.1	1	(−)	D80×	C
3	72	Male	2.1	0.6	(−)	(−)	L
4	58	Male	1.9	1.7	(−)	D80×	A
5	64	Female	1.6	1.5	(−)	D80×	L
6	78	Male	0.8	1.3	(−)	D40×	L
7	48.	Female	1.9	1.5	(−)	D640×	L
8	66	Male	1	0.5	(−)	(−)	L
9	43	Male	1.8	0.7	(−)	N160×	A
10	71	Male	1.1	1	(−)	SP40×	L
11	70	Male	1.9	0.8	(−)	D80×	A
12	60	Male	1.4	1.1	(−)	D40×	A
13	64	Male	1.1	0.9	(−)	SP40×	L
14	49	Male	2.1	0.9	(−) LA +	(−)	A
15	58	Male	1.5	1.4	(−)	D+N40×	A
16	68	Female	1.5	1.2	(−)	D160×	L
17	53	Female	8.1	1.1	(−)	SP N80×	A
18	72	Female	1.5	1	(−)	SP80×	L
19	71	Male	1	0.7	(−)	(−)	L
20	73	Female	1.3	0.8	(−)	SP80×	L
21	64.	Female	1.1	1.3	(−)	D640×	A
22	81	Male	1	0.9	(−)	SP40×	L
23	76.	Male	1.3	1.6	(−)	SP40×	A
24	80	Male	1	1.3	(−)	D80×	A
25	83	Female	0.7	1.4	(−)	(−)	A
26	44	Male	2.1	1.3	(−)	D2560×	C
27	52.	Male	2.1	2.9	(−)	SP40×	A
28	79	Male	0.7	1.1	(−)	(−)	L
29	76	Female	0.8	1.1	(−)	D80×	L
30	65	Male	0.8	1.1	(−)	SP40×	L
31	63.	Female	0.5	1.8	(−)	SP40×	C
32	31	Male	1.4	1.3	(+) LA +	D320×	A
33	50	Male	0.5	1.1	(+) LA +	(−)	A
34	46	Female	5.2	7.6	(−)	SP80×	A

L: lacunar infarction, A: atherothrombotic infarction, C: cardiogenic embolism D: diffuse pattern, SP: speckled pattern, and N: nucleolar pattern.

**Table 3:** Carotid artery echography findings of PI or PS positive patients.

PS or PI positive patients		*P* values
IMT *≧* 1.1 mm	48%	*P* < .05
IMT < 1.1 mm	23%	
Plaque positive	48%	*P* < .05
Plaque negative	21%	
Carotid artery stenosis *≧*50%	62%	*P* < .05
Carotid artery stenosis <50%	31%	

IMT: intimal-medial thickness (*n* = 34)
